# Delayed onset of mediastinitis with tracheomediastinal fistula following endobronchial ultrasound‐guided transbronchial needle aspiration; A case report

**DOI:** 10.1111/1759-7714.13888

**Published:** 2021-02-19

**Authors:** Jong Geol Jang, June Hong Ahn, Seok Soo Lee

**Affiliations:** ^1^ Division of Pulmonology and Allergy, Department of Internal Medicine, College of Medicine, Yeungnam University and Regional Center for Respiratory Diseases Yeungnam University Medical Center Daegu South Korea; ^2^ Department of Thoracic and Cardiovascular Surgery, College of Medicine Yeungnam University and Yeungnam University Medical Center Daegu South Korea

**Keywords:** endobronchial ultrasound, fistula, Mediastinitis, transbronchial needle aspiration

## Abstract

Infectious complications after endobronchial ultrasound‐guided transbronchial needle aspiration (EBUS‐TBNA) are rare but serious. Here, we report a very rare case of delayed onset of mediastinitis with tracheomediastinal fistula after EBUS‐TBNA. Surgical debridement was performed, antibiotics were administered, and the postoperative course of the patient was good. Careful monitoring is needed to prevent the possible development of infectious complications after EBUS‐TBNA.

## Graphical abstract

We report a rare case of delayed onset of mediastinitis with tracheomediastinal fistula after endobronchial ultrasound‐guided transbronchial needle aspiration (EBUS‐TBNA). To our knowledge, tracheomediastinal fistula after EBUS‐TBNA is rarely reported. Surgical debridement was performed, antibiotics were administered, and the postoperative course of the patient was good.

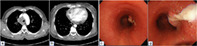

## INTRODUCTION

Endobronchial ultrasound‐guided transbronchial needle aspiration (EBUS‐TBNA) is a minimally invasive technique for the staging of lung cancer and diagnosing mediastinal lymphadenopathy.^1^, ^2^ Infectious complications after EBUS‐TBNA are rare, but they can be fatal if prompt surgical management is not undertaken.^3^, ^4^ Here, we present a very rare case of delayed onset of mediastinitis with tracheomediastinal fistula after EBUS‐TBNA.

## CASE REPORT

**FIGURE 1:**
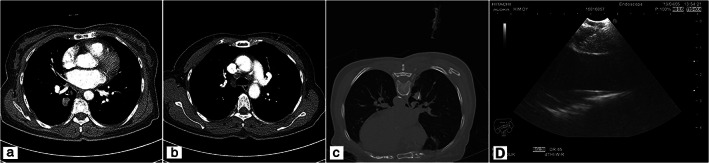
(a, b) Computed tomography (CT) scan showed a solitary pulmonary nodule in the right lower lobe and enlarged right lower paratracheal lymph nodes. (c) Computed tomography (CT)‐guided transthoracic needle biopsy. (d) Endobronchial ultrasonography‐guided transbronchial needle aspiration (EBUS‐TBNA)

A 69‐year‐old non‐smoking woman visited our clinic due to a solitary pulmonary nodule. She had a history of type 2 diabetes mellitus and hypertension. Chest computed tomography (CT) showed a tumor in the right lower lobe with enlargement of the right lower paratracheal lymph nodes (LNs) (Figure 1(a),(b)). CT‐guided percutaneous transthoracic needle biopsy of the nodule was performed using a 20‐gauge cutting needle (Stericut; TSK Laboratory). The EBUS finding of right paratracheal LN revealed a 1 cm sized, oval‐shaped, homogenous echogenicity without necrosis or any cystic findings and there were no abnormal findings on Doppler ultrasound. EBUS‐TBNA with two needle passes were performed to obtain samples from the right lower paratracheal LN using a linear array ultrasonic bronchoscope (PENTAX EB‐1970UK) and single‐use 22‐gauge aspiration needle (MediGlobe, SonoTip EBUS Pro Flex needles) (Figure 1(c),(d)). Biopsy results showed few atypical cells in the right lower lobe and anthracosis without necrosis in the right lower paratracheal LN.

**FIGURE 2:**
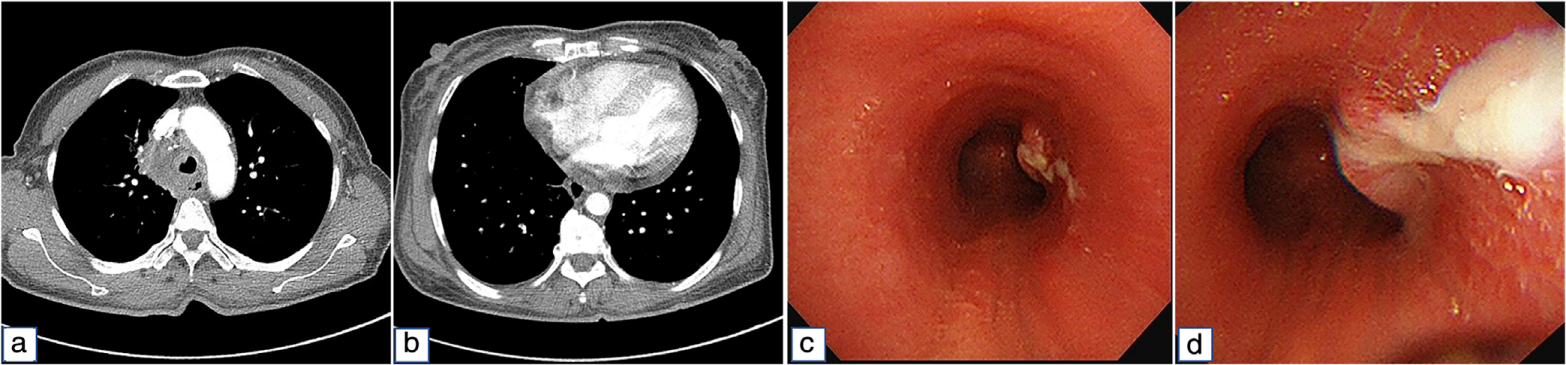
(a, b) Computed tomography (CT) revealed enlargement of mediastinal lymph nodes (a) and pericardial effusion (b), suggestive of mediastinitis. (c, d) Pus draining through the fistula opening in the right lateral aspect of the distal trachea during bronchoscopy

Six weeks later, the patients visited our hospital with a productive cough and history of fever for three days. On admission, her body temperature was 38.2°C; blood tests revealed elevated white cell count (11 720/mm^3^) and C‐reactive protein level (6.524 mg/dl; reference: 0–0.5 mg/dl). A chest radiograph revealed a newly‐developed right paratracheal opacity. Chest CT revealed enlarged mediastinal LNs and pericardial effusion suggestive of mediastinitis (Figure 2(a),(b)). We additionally performed bronchoscopy since the tracheal lumen was determined to be narrowed on CT scan (Figure 2(a)). During flexible bronchoscopy, pus draining through the fistula opening was observed in the right lateral aspect of the distal trachea (Figure 2(c),(d)). Mediastinitis with tracheomediastinal fistula was diagnosed and piperacillin/tazobactam administration along with surgical debridement was performed. After opening the mediastinal pleura via right open thoracotomy, pus was drained from the mediastinum including the paratracheal, subcarinal, and hilar LNs. Gram stain and culture (aerobic and anaerobic) of drained pus resulted in no bacterial growth. Two weeks after surgery, a follow‐up bronchoscopy was performed which confirmed that the fistula opening had closed. The postoperative course was good and the patient was discharged 15 days after hospitalization.

As this study was a clinical case report, no ethics committee approval was required. The patient consented to the publication of the clinical details and images of her case for the purpose of scientific research.

## DISCUSSION

EBUS‐TBNA has previously been used as an initial test for LN staging of lung cancer.^1^, ^2^ A systemic review previously reported that EBUS‐TBNA is safe and has a high diagnostic yield.^5^ However, as the number of cases of EBUS‐TBNA increases, reports on its complications are also increasing.^2^ The complications of EBUS‐TBNA include infectious complications (mediastinitis, pericarditis, and pneumonia), pneumothorax, hemorrhage, and pneumomediastinum.^6^ Among these complications, infectious complications can be serious and require surgical treatment.^3^, ^7^, ^8^, ^9^ They are associated with a longer duration of hospitalization and delayed initiation of subsequent anticancer treatment after diagnosis.^10^, ^11^ Infectious complications after EBUS TBNA are rare; a systematic review and a national survey previously reported the rate of infectious complications was 0.02%–0.19%.^4^, ^12^, ^13^

The pathophysiology of mediastinitis is unclear. Haas hypothesized that infection develops due to direct inoculation of oropharyngeal pathogens to the target lesion by the aspiration needle that is contaminated as it passes through the channel of the bronchoscope.^14^

Risk factors for mediastinitis after EBUS‐TBNA remain unclear. In studies of endoscopic fine needle aspiration, cystic or necrotic lesions were associated with postoperative infectious complications.^15^ However, as in our case, mediastinitis occurred in some cases, even when the lesions were noncystic and non‐necrotic.^11^ The number of samples obtained by EBUS TBNA for each LN and unskilled operator can also be possible risk factors for mediastinitis.^3^ Further research is needed to detect the risk factors for infectious complications.

Despite variability in the timing of mediastinitis onset after EBUS‐TBNA, most cases of mediastinitis occurred within three weeks after EBUS‐TBNA^3^, ^7^, ^8^, ^9^ and the median time of mediastinitis onset in 13 cases was 12.5 days.^11^ One study reported delayed onset in two cases, in whom mediastinitis occurred in patients 40 and 53 days after EBUS‐TBNA.^11^ Our case also showed delayed onset of mediastinitis; thus, possible development of mediastinitis should be investigated. even when a considerable time has elapsed after EBUS‐TBNA.

There is no clear evidence of the efficacy of prophylactic antibiotics to prevent infectious complications after EBUS‐TBNA. A previous preliminary prospective study did not show any efficacy of antibiotic prophylaxis after EBUS‐TBNA.^16^ A study that evaluated the risk factors of infectious complications after EBUS‐TBNA also did not show any benefit of prophylactic antibiotics.^10^ Guidelines for endoscopic ultrasound‐fine needle aspiration recommend antibiotic prophylactic use to obtain cystic lesion samples.^17^ Thus, prophylactic antibiotics might be considered for EBUS‐TBNA of cystic or necrotic lesions. Prophylactic antibiotics that are effective against pathogens found in the oral cavity should be administered since these pathogens have been frequently identified as the cause of mediastinitis after EBUS‐TBNA.^11^ In addition, oral care should be performed before the procedure in order to decrease the risk of infection due to oral cavity pathogens.

In conclusion, here we present a very rare case of delayed onset of a potentially fatal infectious complication after EBUS‐TBNA. Unlike previously reported cases with onset of mediastinitis within three weeks, our case showed a delayed onset (six weeks) of mediastinitis with coexisting tracheomediastinal fistula. With the increasing number of EBUS‐TBNA, careful monitoring is needed to prevent possible infectious complications.

## ACKNOWLEDGEMENTS

This study was supported by the Yeungnam University Research Fund for 2020.

## CONFLICT OF INTEREST

The authors declare there are no conflicts of interest.
